# Volumetric breast density estimation on MRI using explainable deep learning regression

**DOI:** 10.1038/s41598-020-75167-6

**Published:** 2020-10-22

**Authors:** Bas H. M. van der Velden, Markus H. A. Janse, Max A. A. Ragusi, Claudette E. Loo, Kenneth G. A. Gilhuijs

**Affiliations:** 1 Image Sciences Institute, University Medical Center Utrecht, Utrecht University, Q.02.4.45, P.O. Box 85500, 3508 GA Utrecht, The Netherlands; 2 Department of Radiology, The Netherlands Cancer Institute, Antoni van Leeuwenhoek Hospital, Amsterdam, The Netherlands

**Keywords:** Breast cancer, Risk factors, Biomarkers

## Abstract

To purpose of this paper was to assess the feasibility of volumetric breast density estimations on MRI without segmentations accompanied with an explainability step. A total of 615 patients with breast cancer were included for volumetric breast density estimation. A 3-dimensional regression convolutional neural network (CNN) was used to estimate the volumetric breast density. Patients were split in training (N = 400), validation (N = 50), and hold-out test set (N = 165). Hyperparameters were optimized using Neural Network Intelligence and augmentations consisted of translations and rotations. The estimated densities were evaluated to the ground truth using Spearman’s correlation and Bland–Altman plots. The output of the CNN was visually analyzed using SHapley Additive exPlanations (SHAP). Spearman’s correlation between estimated and ground truth density was ρ = 0.81 (N = 165, P < 0.001) in the hold-out test set. The estimated density had a median bias of 0.70% (95% limits of agreement = − 6.8% to 5.0%) to the ground truth. SHAP showed that in correct density estimations, the algorithm based its decision on fibroglandular and fatty tissue. In incorrect estimations, other structures such as the pectoral muscle or the heart were included. To conclude, it is feasible to automatically estimate volumetric breast density on MRI without segmentations, and to provide accompanying explanations.

## Introduction

Breast density refers to the amount of fibroglandular tissue with respect to the fatty tissue. It is a well-known risk factor for the development of breast cancer^1^, and is incorporated in several breast cancer risk models^2,3^. Most states in the United States of America require reporting of breast density^4^.

Breast density can be assessed on imaging such as mammography and magnetic resonance imaging (MRI). In clinical practice, radiologists score breast density in one of four incremental categories: almost entirely fatty, scattered fibroglandular tissue, heterogeneously dense, or extremely dense^5^.

Breast density can also be quantified using computer algorithms. Such algorithms have been investigated both for mammography and MRI, and show strong correlation between the two modalities^6,7^. On MRI, these methods typically consist of 3-dimensional segmentation of the breast region and the fibroglandular tissue^8–14^. The volumetric density is then defined as the volume of the fibroglandular tissue divided by the volume of the breast region. In these studies, the average Dice similarity coefficient for the segmented fibroglandular tissue is roughly 0.8, and can be as low as 0.6.

In case of incorrect segmentation, manual correction by a radiologist is possible. This may take several minutes in case of minor corrections, up to about an hour in case of full segmentation^9^. Furthermore, automatic checking of these segmentations is challenging in a clinical setting, since the ground truth segmentation is lacking.

There is a need to skip the segmentation for breast density estimation, but still give insight in the estimation that can easily be checked and corrected by a radiologist. The aim of this paper is to assess the feasibility of volumetric breast density estimations on MRI without segmentations accompanied with an explainability step.

## Material and methods

### Patients

Data were acquired after written informed patient consent and after approval of the Medical Ethics Committee of The Netherlands Cancer Insitute-Antoni van Leeuwenhoek hospital in accordance with the declaration of Helsinki^15^. We performed a post-hoc analysis of the prospective Multimodality Analysis and Radiological Guidance IN breast conServing therapy study (MARGINS, 2000–2008), that was performed at The Netherlands Cancer Insitute-Antoni van Leeuwenhoek hospital^16,17^. In the MARGINS study, patients who were eligible for breast conserving surgery based on conventional imaging and clinical examination were consecutively recruited for a preoperative breast MRI. Proof of breast cancer was acquired using core biopsy or fine-needle aspiration and surgery.

**Figure 1:**
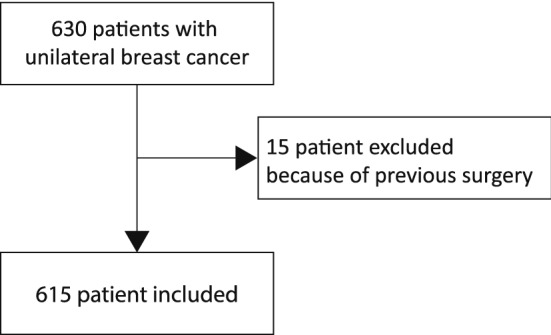
Flowchart of patient inclusion.

We included all patients with unilateral breast cancer (N = 630) to estimate volumetric breast density in the unaffected breast. Patients who received previous surgery in the contralateral breast were excluded (N = 15/630, 2%). Finally, 615/630 (98%) patients are included in the analysis (Fig. 1).

### Magnetic resonance imaging

The patients were imaged using a 1.5T MRI unit (Magneton, Siemens) with a dedicated double-breast array coil (CP Breast Array, four channels, Siemens). Dynamic contrast-enhanced series were acquired using fast low-angle shot 3-dimensional T1-weighted imaging, reconstructed to isotropic voxel size of 1.35 × 1.35 × 1.35 mm^3^. The imaging parameters were: repetition time 8.1 ms, echo time 4.0 ms, flip angle 20°, and field of view 310 mm, without fat suppression. We used the precontrast image of the contrast-enhanced series for density estimation.

### Ground truth creation

To determine the ground truth volumetric breast density, we automatically extracted the breast region and the fibroglandular tissue region in 3-dimensions from the precontrast images of the unaffected breast^18^. In short, the breast area was segmented adopting a custom-developed knowledge-based breast segmentation tool^19^. The fibroglandular tissue was subsequently segmented in the breast area segmentation using fuzzy c-means clustering^20^. We manually checked and, if necessary, corrected these segmentations. The posterior cutoff of the breast region was chosen at the sternum^10^. The volumetric density was defined as the number of voxels in the fibroglandular tissue segmentation divided by the number of voxels in the breast region segmentation times 100%^18^. This volumetric density was subsequently used as the ground truth, hence yielding a single label per MRI. The previously described segmentations were not used in any way for further analysis.

### Image preprocessing

We removed field inhomogeneities from the MR images using N4 bias field correction^21^. We normalized the MR image intensities between zero and one based on the 2.5th and 97.5th intensity percentiles^22^. Voxels outside that range were clipped.

### Volumetric density estimation

**Figure 2:**
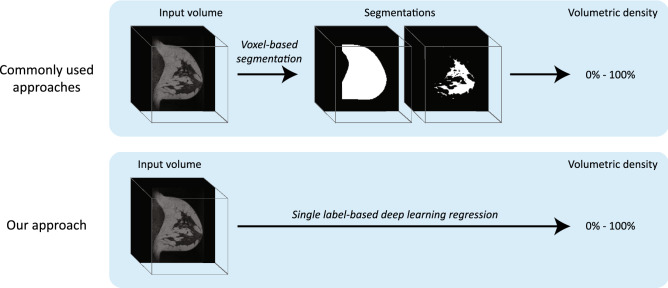
The difference between our approach and other commonly used approaches. Commonly used approaches typically first segment the volumes of interest—i.e. the breast volume and the fibroglandular tissue volume—and then divide the two to obtain a volumetric density measure. Our approach directly assesses the volumetric density by learning the relation between a 3-dimensional volume and a single label—i.e. the volumetric density. As illustration, the 3-dimensional volumes are depicted as a cube with an insert of a 2-dimensional sagittal slice.

**Figure 3:**
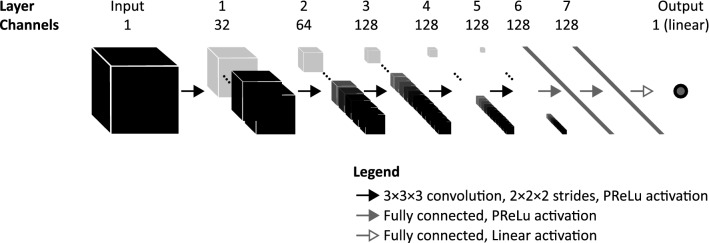
The convolutional neural network (CNN) architecture. The input of the CNN consisted of 3-dimensional volumes of 128 × 128 × 128 voxels. These volumes contained the contralateral breasts of the patients. The architecture of the CNN consists of five convolutional layers, two fully connected layers consisting of 128 nodes, and a linear activation as output.

We used a 3-dimensional regression convolutional neural network (CNN) to directly estimate the volumetric breast density from the MR image (illustrated in Fig. 2). The input of the CNN consisted of 3-dimensional volumes of 128 × 128 × 128 voxels. These volumes contained the contralateral breasts of the patients. The architecture of the CNN consisted of five convolutional layers, two fully connected (dense) layers consisting of 128 neurons each, and a linear activation as output^23^ (Fig. 3). The five layers consisted of a kernel size of 3 × 3 × 3, a stride of 2 × 2 × 2, parametric rectified linear unit activations^24^, and 50% dropout^25^. The number of convolutional filters doubled in the second and third layer. We used mean absolute error as loss function and the Adam optimizer^26^. The patients were split on ranked study number into a training set (N = 400), a validation set (N = 50), and a hold-out test set (N = 165).

We optimized the hyperparameters on the validation set with the Neural Network Intelligence toolkit (Microsoft, Redmond, WA, USA). The search space was: starting number of convolutional filters between 4 and 32, learning rate between 0.01 and 10^–8^. Augmentations consisted of random translations in the left–right and superior-inferior directions (maximum translation of 20 voxels) and random rotations along the sagittal and transversal axes (maximum rotation of 10 degrees).

We performed the deep learning experiments using an NVIDIA GeForce RTX2080 Ti GPU.

### Statistical evaluation

We statistically evaluated potential biases in the patient baseline characteristics and the volumetric density between train set, validation set, and hold-out test set. For this purpose we used the nonparametric Kruskall–Wallis test for continuous values and the χ^2^ test for categorical values.

We evaluated the deep learning algorithm by comparing the volumetric density estimations of the CNN to the ground truth using Spearman correlations coefficient. In addition, we estimated the bias and variance of the algorithm using nonparametric Bland–Altman plots^27^.

As a subanalysis, we investigated the Spearman correlation coefficient between the volumetric density estimations of the CNN and the ground truth separately for non-dense breast and for dense breast. Since we did not have clinical density (i.e. the BI-RADS density scores^5^), we used a volumetric breast density of 15.5% as threshold between non-dense and dense breasts^7^.

### Algorithm explanations

We used Deep SHapley Additive exPlanations (SHAP) to explain the output of the algorithm^28^. In short, SHAP is a method to explain a prediction by computing the contribution of each feature to that prediction. It does so using Shapley values^29^. Shapley values ensure a ‘fair’ division of attribution: the marginal contribution of every voxel to the final volumetric density estimation is taken into account individually^29^. These contributions can be positive and negative.

For each patient, Deep SHAP yields a map of SHAP-values. Each voxel in this SHAP-values map represents the contribution of that pixel to the final decision. Hence, higher values correspond to higher volumetric density estimations, and lower values correspond to lower volumetric density estimations.

To assess the background signal needed for the Deep SHAP analysis^28^, we randomly sampled 15 precontrast volumes from the training set.

## Results

### Patients

**Figure 4:**
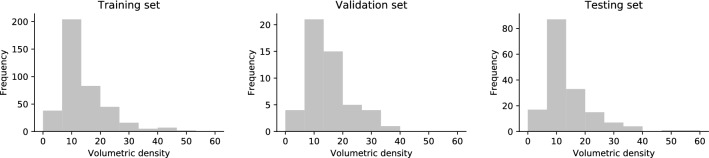
Distributions of volumetric densities in the train set (N = 400), the validation set (N = 50) and the hold-out test set (N = 165).

The baseline characteristics (Table 1) and the volumetric density were not significantly different between train, validation, and hold-out test set (P > 0.20, Fig. 4), confirming an unbiased split.

**Table 1:** Baseline characteristics of the included patients (N = 615).

Variable	Value
Age at diagnosis in years, median (range)	57 (26–86)
Largest tumor diameter on MRI in mm, median (range)	19 (5–90)
**Immunohistochemical subtype**	
ER + HER2-	438 (71)
HER2 +	79 (13)
TN	77 (13)
Unknown	21 (3)
**Histological finding**	
Invasive ductal carcinoma	444 (72)
Invasive lobular carcinoma	81 (13)
Other invasive carcinoma	33 (5)
Mixed invasive pattern	11 (2)
Ductal carcinoma in situ	37 (6)
Lobular carcinoma in situ	1 (0)
Unknown	8 (1)
**Histological grade**	
Grade I	191 (31)
Grade II	259 (42)
Grade III	151 (25)
Unknown	14 (2)
**Axillary load**	
no positive lymph nodes	396 (64)
1–3 positive lymph nodes	165 (27)
4 or more positive lymph nodes	35 (6)
Unknown	19 (3)

Values are counts (percentages) unless otherwise stated.

### Volumetric density estimation

**Figure 5:**
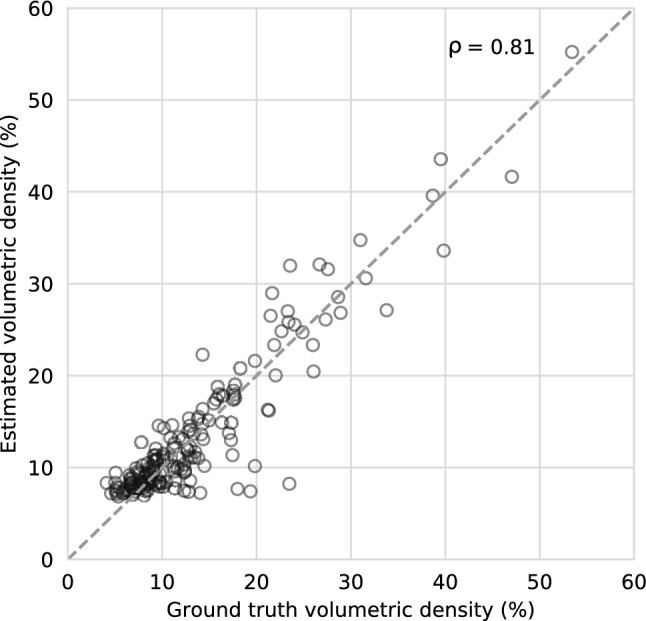
Correlation between estimated volumetric density and ground truth volumetric density in the hold-out test set (N = 165, Spearman’s ρ = 0.81, P < 0.001).

The Spearman’s correlation between the estimated volumetric density and the ground truth volumetric density was ρ = 0.81 (N = 165, P < 0.001) in the hold-out test set (Fig. 5).

**Figure 6:**
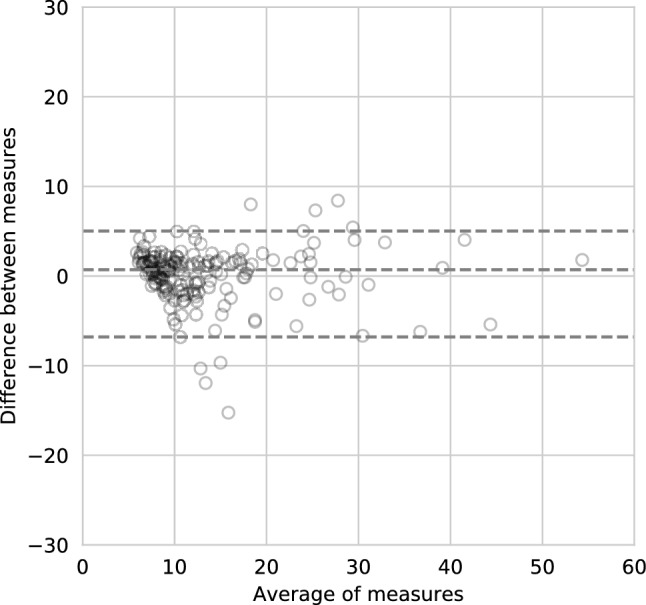
Bland–Altman analysis shows that the estimated volumetric density has a mean bias of 0.70% (95% limits of agreement =  − 6.8% to 5.0%) when compared to the ground truth volumetric density. The x-axis shows the average of the two measures, the y-axis the difference between the two.

Nonparametric Bland–Altman analysis showed that the estimated volumetric density had a median bias of 0.70% (95% limits of agreement = − 6.8% to 5.0%) compared to the ground truth volumetric density (Fig. 6).

The Spearman’s correlation between the estimated volumetric density and the ground truth volumetric density was ρ = 0.69 (N = 117, P < 0.001) in the patients with non-dense breasts, and ρ = 0.80 (N = 48, P < 0.001) in the patients with dense breasts.

### Algorithm explanations

**Figure 7:**
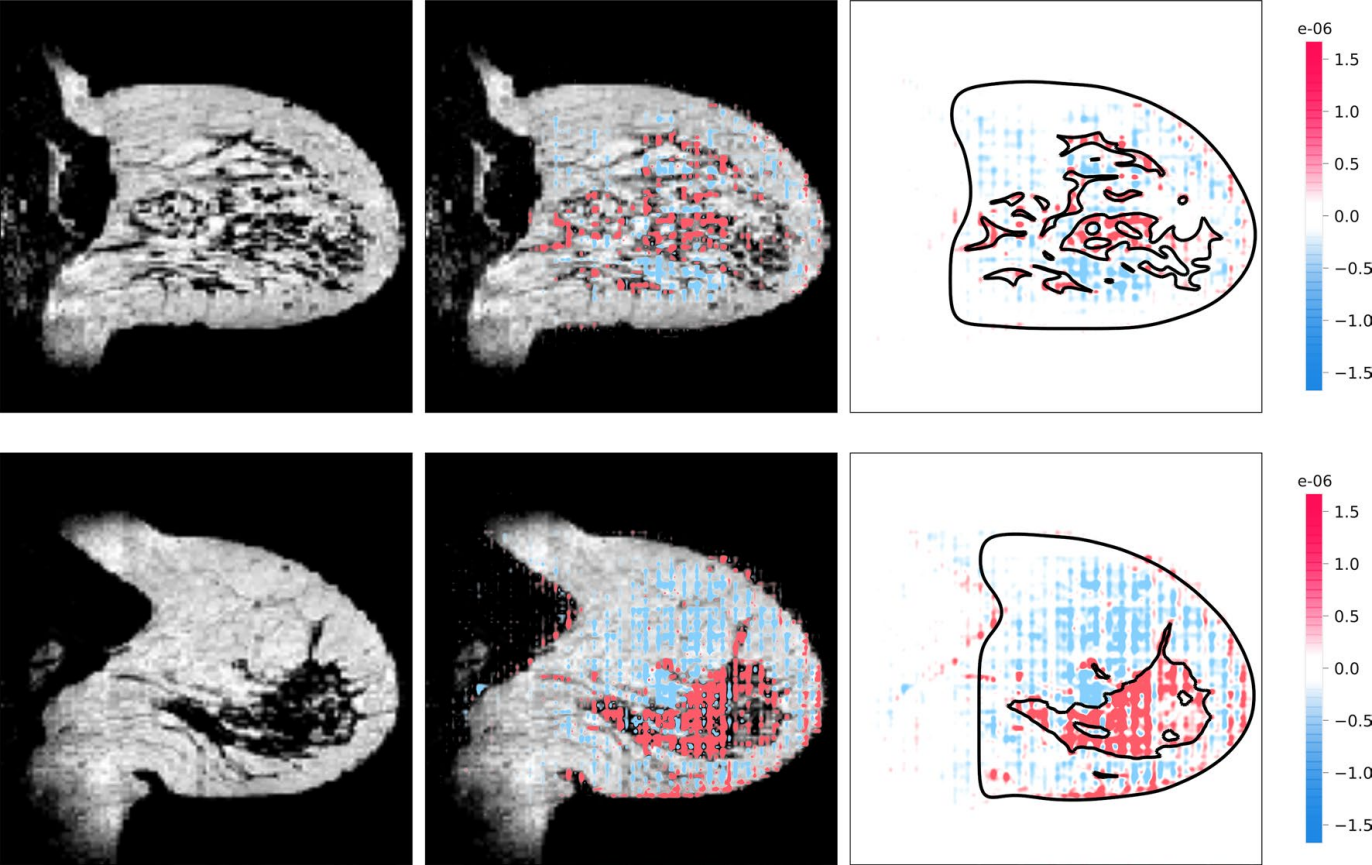
Examples of correct volumetric density estimations from the hold-out test set. For both the top and bottom patient, the left image is a sagittal slice of the precontrast MRI, the middle image is the same sagittal slice with the SHAP-map superimposed, and the right image is the corresponding SHAP-map. For illustrative purposes, the boundaries of the breast and fibroglandular tissue are shown in the black contours. It can be seen in the SHAP-maps that the glandular tissue voxels are responsible for an increase in density estimation (red) and the fatty tissue voxels are responsible for a decreased density estimation (blue). Top: ground truth volumetric density 17.6%, predicted volumetric density 17.9%. Bottom: ground truth volumetric density 8.5%, predicted volumetric density 9.3%. Although the density is based on a 3-dimensional volume, a single sagittal slice is shown for illustrative purposes.

**Figure 8:**
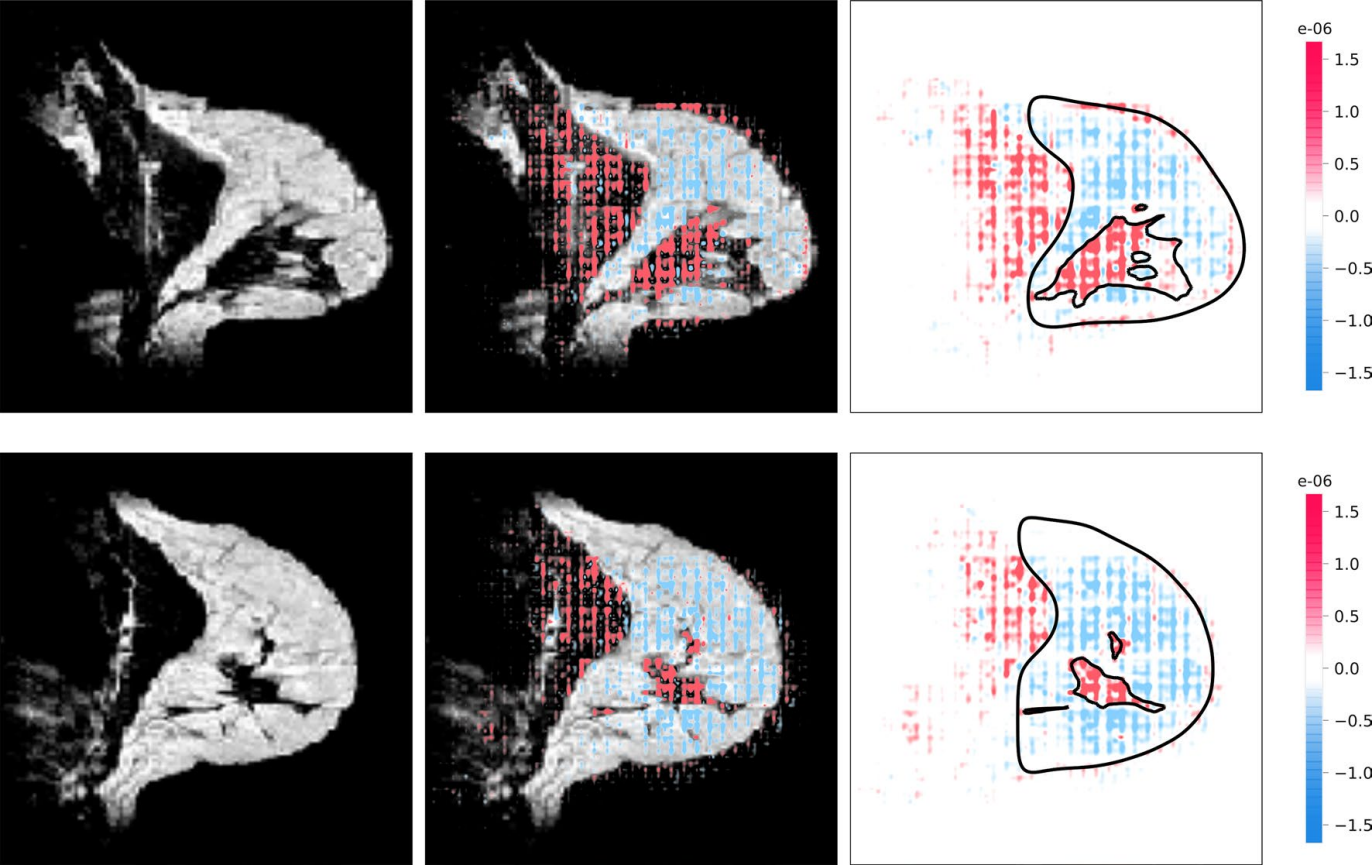
Examples of incorrect volumetric density estimations from the hold-out test set. For both the top and bottom patient, the left image is a sagittal slice of the precontrast MRI, the middle image is the same sagittal slice with the SHAP-map superimposed, and the right image is the corresponding SHAP-map. For illustrative purposes, the boundaries of the breast and fibroglandular tissue are shown in the black contours. The SHAP-maps show that the overestimation is based on the pectoral muscle (top and bottom) and part of the heart (bottom). Although the density is based on a 3-dimensional volume, a single sagittal slice is shown for illustrative purposes.

Explanations of the density estimation algorithm showed that in correct volumetric density estimations, the algorithm based its decision on the glandular and fatty tissue (Fig. 7). In incorrect density estimations, it can be seen that the algorithm also based its decision on other anatomical structures, such as the pectoral muscle or the heart (Fig. 8).

## Discussion

We showed the feasibility of a method to automatically estimate volumetric breast density on MRI without using segmentations. The Spearman correlation coefficient between the estimated breast density and the ground truth density was 0.81 in the 165 patients from the hold-out test set. Interpretation of these estimations were provided using Shapley Additive exPlanations (SHAP).

The main advantage of our method is that it does not need costly voxel-level 3-dimensional segmentations of the breast and fibroglandular tissue. Other methods to assess breast density on MRI typically do need these segmentations^11–14^, which can take up to an hour to obtain^9^. Our method only needs a single label per breast, which can easily be obtained with software such as the commercially available Densitas (Densitas Inc, Halifax, NS, Canada), Quantra (Hologic Inc, Bedford, MA, USA), or Volpara (Volpara Health Technologies, Wellington, New Zealand) software. Hence, our proof-of-principle method is easily scalable.

The ground truth volumetric density in this study was based on automatic breast and fibroglandular tissue segmentations. We chose to limit the breast region on the posterior side at the sternum^10^. Choosing a more posterior limit would include the axilla where the signal from the breast coil is influenced by patient anatomy. Hence, this would complicate the cutoff of the breast region, and lead to more variations in ground truth. Other researchers have employed comparable strategy^10^, and the range of volumetric breast densities in our study is comparable to those reported by others^7,30–33^. The fibroglandular tissue segmentations were based on fuzzy c-means^20^. These segmentations have been extensively checked. Although the ground truths are manually confirmed, it is possible to substitute them with other volumetric density measures, such as those provided by the previously mentioned Densitas, Quantra, or Volpara software. The algorithm would work in exactly the same way.

Other methods to establish breast density on MRI often segment the breast region and fibroglandular tissue. These methods include dynamic programming with fuzzy c-means (N = 11, Dice similarity coefficient (DSC) not reported)^8^, a 2-dimensional atlas combined with fuzzy c-means (N = 60, mean DSC of fibroglandular tissue = 0.60)^9^, and an atlas combined with Gaussian mixture modeling (N = 50, mean DSC of fibroglandular tissue = 0.80)^10^. Since the introduction of deep learning in medical image analysis, several 2-dimensional U-net architectures have been proposed (ranging from N = 40 to N = 286, and mean DSCs of fibroglandular tissue from 0.83 to 0.92)^11,13,14^, as well as a 3-dimensional U-Net (N = 137, mean DSC of fibroglandular tissue = 0.81)^12^. These methods rely on manual segmentations or (semi)-automatically derived segmentations. The manual segmentations are costly to acquire, whereas the (semi)-automatic segmentations would need to be checked by an experienced observer. The method that we discuss in this paper only uses the volumetric density measure. Therefore, we do not need to have segmentation-based ground truth annotations, making our proof-of-principle study easily scalable.

The algorithm provides a density score. When a radiologist does not trust the density score, the radiologist can assess the estimation using the explanations provided by the Shapley Additive exPlanations. A potential future workflow could be to assess these explanations, and if the estimation is based on incorrect regions—such as the pectoral muscle—exclude those regions from the classifier’s decision.

We chose Shapley Additive exPlantions, because it uses Shapley values which ensure a ‘fair’ division of attribution. This means that the marginal contribution of every voxel to the final volumetric density estimation is taken into account individually. Therefore, these contributions of the voxels are assessed optimally, a property that is not necessarily present in other techniques that consider local image regions such as LIME (local interpretable model-agnostic explanations)^34^. A pitfall of Shapley-values is that they are very costly to compute, since they require assessment of many permutations. By using the implementation of Deep SHAP, the computation time is acceptable (in our case a second per 3-dimensional volume), whilst still maintaining the abovementioned properties of the Shapley values^28^.

We chose our network architecture based on a previous study using regression for 2-dimensional slice selection in chest CT imaging^23^ and expanded it from 2-dimensional to 3-dimensional. Other network architectures could be investigated in future work. Our paper has several strengths. The most notable strength is that our algorithm works with a single label per patient. Although in the current study this label was acquired using previously generated segmentations, the label could also be acquired from other systems, such as the commercially available Volpara Density for mammography. Therefore, expanding the training set for the current system is relatively cheap, just one density label needs to be provided, not an entire breast and fibroglandular tissue segmentation. Another strength is that our algorithm works fully on 3-dimensional data.

The most notable limitation of our study is that the system is trained and evaluated on single-institution data from a single MRI vendor. In order to fully elucidate on the potential of the proposed method, multi-institution and multi-vendor data would be desired. The current study does, however, demonstrate the feasibility of the proposed method. Future work will include multi-institutional and multi-vendor validation.

To conclude, we showed that it is feasible to automatically estimate volumetric breast density on MRI without segmentations, and provided accompanying explanations.

## Data Availability

The datasets analyzed during the current study are not publicly available due to patient privacy but are available at the corresponding author on reasonable request.
